# COVID-19-Associated Candidiasis (CAC): An Underestimated Complication in the Absence of Immunological Predispositions?

**DOI:** 10.3390/jof6040211

**Published:** 2020-10-08

**Authors:** Amir Arastehfar, Agostinho Carvalho, M. Hong Nguyen, Mohammad Taghi Hedayati, Mihai G. Netea, David S. Perlin, Martin Hoenigl

**Affiliations:** 1 Center for Discovery and Innovation, Hackensack Meridian Health, Nutley, NJ 07110, USA; david.perlin@hmh-cdi.org; 2 Life and Health Sciences Research Institute (ICVS), School of Medicine, University of Minho, 4710-057 Braga, Portugal; 3 ICVS/3B’s—PT Government Associate Laboratory, 4710-057 Guimarães/Braga, Portugal; 4 Department of Medicine, University of Pittsburgh, Pittsburgh, PA 15261, USA; mhn5@pitt.edu; 5 Invasive Fungi Research Center, Department of Medical Mycology, School of Medicine, Mazandaran University of Medical Sciences, Sari 4815733971, Iran; hedayatimt@gmail.com; 6 Department of Internal Medicine and Radboud Center for Infectious Diseases, Radboud University Medical Centre, 6500HB Nijmegen, The Netherlands; Mihai.Netea@radboudumc.nl; 7 Department of Genomics & Immunoregulation, Life and Medical Sciences Institute, University of Bonn, 53115 Bonn, Germany; 8 Radboud Institute for Molecular Life Sciences, Radboud University Medical Center, 6525 GA Nijmegen, The Netherlands; 9 Clinical and Translational Fungal-Working Group, University of California San Diego, La Jolla, CA 92093, USA; 10 Section of Infectious Diseases and Tropical Medicine, Department of Internal Medicine, Medical University of Graz, 8036 Graz, Austria; 11 Division of Infectious Diseases and Global Public Health, Department of Medicine, University of California, San Diego, San Diego, CA 92093, USA

**Keywords:** candidemia, candiduria, oral candidiasis, mycobiome

## Abstract

The recent global pandemic of COVID-19 has predisposed a relatively high number of patients to acute respiratory distress syndrome (ARDS), which carries a risk of developing super-infections. *Candida* species are major constituents of the human mycobiome and the main cause of invasive fungal infections, with a high mortality rate. Invasive yeast infections (IYIs) are increasingly recognized as s complication of severe COVID-19. Despite the marked immune dysregulation in COVID-19, no prominent defects have been reported in immune cells that are critically required for immunity to *Candida*. This suggests that relevant clinical factors, including prolonged ICU stays, central venous catheters, and broad-spectrum antibiotic use, may be key factors causing COVID-19 patients to develop IYIs. Although data on the comparative performance of diagnostic tools are often lacking in COVID-19 patients, a combination of serological and molecular techniques may present a promising option for the identification of IYIs. Clinical awareness and screening are needed, as IYIs are difficult to diagnose, particularly in the setting of severe COVID-19. Echinocandins and azoles are the primary antifungal used to treat IYIs, yet the therapeutic failures exerted by multidrug-resistant *Candida* spp. such as *C. auris* and *C. glabrata* call for the development of new antifungal drugs with novel mechanisms of action.

## 1. Introduction

Yeast species belonging to the *Candida* genus, including *Candida albicans*, *Candida glabrata*, *Candida parapsilosis*, *Candida tropicalis*, and *Candida krusei*, are the most prevalent fungal species inhabiting various mucosal surfaces, such as the skin and the respiratory, digestive, and urinary tracts [1,2]. Although being commensal within the human host, *Candida* species are equipped with virulence attributes, enabling them to invade when opportunities arise and cause various infections in humans, especially when the immune system is impaired [2]. Superficial infections, such as skin disorders; mucosal infections, including oropharyngeal or vulvovaginitis candidiasis; and invasive candidiasis are established clinical entities of candidiasis [3,4,5,6,7,8]. *Candida* is among the most frequently recovered pathogen in the intensive care unit (ICU), affecting between 6% and 10% of patients, and some studies have noted an increasing trend for candidemia [9]. The estimated mortality attributed to invasive candidiasis is 19–40% [10]. This mortality is even higher among ICU patients, approaching 70% [11]. Apart from being associated with excess economic costs and approximately 1.5 million annual deaths [8], the new landscape of candidemia reveals an increasing incidence of non-*albicans Candida* (NAC) species, with intrinsic resistance to antifungals and/or with a propensity to rapidly acquire antifungal resistance [12]. More troubling is the recent emergence of multidrug-resistant (MDR) *Candida* species, including *C. glabrata* and *C. auris* [13,14,15,16], the increasing trend of fluconazole-resistant *C. parapsilosis* and *C. tropicalis* [13,17], and inherently resistant *C. krusei*, which notoriously affect the efficacy of antifungal treatment.

The recent global pandemic of COVID-19 has resulted in an unprecedented 890,000 deaths worldwide [18]. A notable proportion of COVID-19 critically ill patients develop acute respiratory distress syndrome (ARDS), requiring intensive care unit (ICU) admission and mechanical ventilation, which in turn predisposes them to nosocomial infections due to bacterial and fungal infections [19,20]. Understanding the burden of COVID-19 patients with secondary infections and their etiologic agents is paramount for the optimal management of COVID-19 patients. This knowledge will help to refine empiric antimicrobial management for patients with COVID-19 with the hope to improve patient outcomes.

Despite the recognition that airborne *Aspergillus fumigatus* is increasingly recognized as an important cause of fungal super-infections among critically ill COVID-19 patients [19], the incidence of candidiasis has not been evaluated in this context. Indeed, the wide use of antibiotics, corticosteroids, and central venous catheters, along with the damage exerted by SARS CoV-2 among patients with ARDS [19], may allow commensal *Candida* to cells to invade internal organs [20,21,22,23,24,25,26,27]. The goals of this manuscript are to review our current knowledge on *Candida* super-infections among COVID-19 patients, discuss the potential immunological and clinical factors predisposing these patients to invasive candidiasis, and outline what studies are needed to better define the epidemiology of this superinfection.

## 2. Immunology

### 2.1. General Pathophysiology of SARS COV-2

Similar to other SARS coronaviruses, the pathophysiology of SARS-CoV-2 involves targeting and invading epithelial cells and type II pneumocytes through the binding of the SARS spike protein to the angiotensin-converting enzyme 2 (ACE2) receptor [28]. During the course of the host–virus interaction, the type 2 transmembrane protease TMPRSS2 cleaves the S1/S2 domain of the viral spike protein [29] and promotes viral entry into the target cells. ACE2 is required for protection from severe acute lung injury in ARDS [30], and the viral-mediated manipulation of this receptor is considered one major mechanism contributing to severe lung injury in selected COVID-19 patients. The degree of variability in the severity of disease is also supported, at least in part, by the existence of genetic variants that affect the ACE2 activity and underlie an increased susceptibility to ARDS and worse prognosis [31]. Besides the implications of ACE2 in the pathogenesis of COVID-19, recent studies have also suggested that the disruption of the renin-angiotensin system and/or the kallikrein-kinin system could contribute to the detrimental inflammatory phenotype observed in patients with severe COVID-19 [32,33].

### 2.2. Does Immunity Renders Susceptibility to Invasive Yeast Infections?

Infection with SARS-CoV2 elicits an immune response that triggers an inflammatory cascade as the result of the activity of innate immune cells. However, the dynamics of how the immune system senses and responds to SARS-CoV-2 is just unfolding, which limits our understanding of possible immune-mediated pathways contributing to the pathogenesis COVID-19-associated candidiasis (CAC). Cell types important for host defense against *Candida*, such as neutrophils and monocytes/macrophages, are not affected by SARS-CoV-2, suggesting that they are not responsible for CAC. Indeed, single-cell analyses of bronchoalveolar lavages from critically ill patients with COVID-19 showed an abundance of monocyte-derived macrophages [34]. Similarly, an increased peripheral neutrophil-to-lymphocyte ratio was also observed in severe cases of COVID-19, and was likely associated with unfavorable prognosis [35]. While the increasing numbers (and activation profiles) of these cells may contribute to tissue damage and the severity of disease, they are an unlikely risk factor for invasive candidiasis. One exception is the decreased expression of human leukocyte antigen DR on the membrane of circulating monocytes [36], which is considered a marker of immune paralysis; however, its relevance in susceptibility to candidemia is unclear. The clear immune defect in patients with COVID-19 is, on the other hand, lymphopenia; however, an isolated decrease in lymphocyte numbers, as also experienced by HIV patients, is not associated with an increase in susceptibility to systemic *Candida* infections. Taken together, these findings support the concept that classical risk factors for invasive candidiasis, rather than an overt immune dysfunction, are the major drivers of susceptibility to CAC.

## 3. Epidemiology of CAC, Clinical and Microbiological Factors: Current Paradigm

To obtain studies reporting yeast infections among patients with COVID-19, we included all studies published up to September 10, 2020. Our search included yeast, *Candida*, COVID-19, fungal super-infection +COVID-19, and fungal super-infections + COVID-19, and we used both the Google and PubMed search engines. The extent of CAC (both superficial and invasive) varies by country and region. Studies from Spain [37], India [27], Iran [22], Italy [26], the UK [23], and China [20] have reported rates of 0.7% (7/989), 2.5% (15/596), 5% (53/ 1059), 8% (3/43), 12.6% (17/135), and 23.5% (4/17) [20], respectively (Table 1). Although a previous study from Iran indicated a relatively low level of oral candidiasis (OC) among patients with COVID-19 (53/1059), apparently that study included all the patients who presented with COVID-19 but not those developing ARDS, which may have resulted in an underestimation of OC in the context of COVID-19 [22].

A study from Spain reported a rate of 0.7% (7/989) of fungal super-infections complicating hospitalized COVID-19 patients: four were caused by molds and three by *Candida* (one each of candidemia, candiduria, and complicated intraabdominal candidiasis (IAC)) [37]. Similarly, a recent study from the UK reported a similar prevalence of invasive yeast infections and invasive pulmonary aspergillosis (12.6% vs. 14.1%) among COVID-19 patients who presented with ARDS [23]. A study from Greece reported that two COVID-19 patients residing in an ICU developed bloodstream infection due to *Saccharomyces cerevisiae* a few days (4–6 days) after receiving a probiotic supplement (Ultra-Levure) which contains this yeast. Interestingly, none of the 320 patients admitted to the same unit in the previous 4 years developed *S. cerevisiae* fungemia while receiving the same probiotic [21]. This observation, while anecdotal, suggests that the sepsis syndrome or septic shock associated with severe COVID-19 may damage the intestinal mucosal barrier, enabling the translocation of concentrated fungus in probiotics (250–500 mg/day in this case), leading to fungemia [38,39]. This study cautions about the routine use of prophylactic probiotics among critically ill COVID-19 cases in the ICU setting.

Moreover, a recent study from Italy also reported three candidemia cases among critically ill COVID-19 patients following treatment with tocilizumab, an IL-6 receptor monoclonal blocking agent [26]. Central venous catheterization (CVC) (32/43; 74.5%), antibiotics (26/43; 60.5%), and steroid therapy use (13/43; 13.2%) were among the most prominent risk factors reported (Table 1). Overall, the mortality rate of invasive *Candida* infections was approximately 46% (20/ 43), which varied depending on the species and the antifungal used to treat invasive yeast infections. Indeed, this mortality rate is presumably higher than that of severely ill patients with COVID-19, ranging between 25.8% [40] and 30.9% [41]. Per species, the mortality rate was the highest for patients infected with *C. glabrata* (2/2; 100%), *C. auris* (6/10; 60%), and *C. albicans* (8/19; 42%), while those treated with *C. tropicalis*, *C. parapsilosis*, and multiple *Candida* species all survived (two patients infected with *C. krusei* and *Rhodotorula* spp. and two with unknown species also died). It is noteworthy that those results may be misleading due to the limited numbers, since *C. tropicalis* has been shown before to be associated with the poorest prognosis and also carries a high rate of mortality when compared to other non-*Candida albicans Candida* species [42,43].

According to recent studies detailing invasive yeast infections among critically ill COVID-19 patients (21, 23–27), *C. albicans* (19/43; 44.1%) was shown to be the most prevalent yeast species, followed by *C. auris* (10/43; 23.2%); *C. glabrata*, *C. parapsilosis*, *C. tropicalis*, and *S. cerevisiae* (2/43; 4.6% each); and *C. krusei* and *Rhodotorula* spp. (1/43; 2.3% each). Of note, there was no species identity reported for two yeast isolates obtained from catheter and chest drain, and two patients had mixed invasive yeast infections caused by *C. albicans* + *C. parapsilosis* and *C. albicans* + *C. tropicalis* (Table 1). Importantly, *C. auris* was the most prevalent *Candida* species from the Indian study, while *C. albicans* was the most prevalent in the other studies. Where antifungal susceptibility testing was performed, the resistance patterns varied depending on the species. For instance, resistance to fluconazole, multiple azoles (fluconazole and voriconazole), and multidrugs (fluconazole and AMB) was noted for 100%, 30%, and 40% of the *C. auris* isolates, respectively, and only one *C. glabrata* isolate was echinocandin-resistant (Table 1). Persistent invasive yeast infections have been noted during the course of antifungal therapy, while the yeasts isolated showed susceptible profiles to the antifungal used for treatment [25,27]. Most notably, 67% of the patients who died with invasive yeast infections due to *C. auris* showed persistent candidemia, despite being treated with micafungin [27] in the absence of resistance, which might be explained by other host and pathogen-related factors [44,45,46]. Therefore, these data highlight the urgency of conducting comprehensive studies elucidating the real burden of each entity among COVID-19 cases manifesting ARDS.

## 4. Risk Factors

The risk factors for CAC can be divided into two groups. The first group includes common risk factors predisposing ICU patients to invasive candidiasis. These include diabetes mellitus, renal failure requiring hemodialysis, abdominal surgery, triple lumen catheters, parenteral nutrition, receipt of multiple antibiotics, length of ICU stay >7 days, and prior abdominal infections [10,47,48]. Additionally, indwelling central venous catheters are widely used among COVID-19 patients residing in ICUs [49]. Indeed, catheters are historically known as a portal of entry for acquiring nosocomial *Candida* infections, such as *Candida auris* and *C. parapsilosis* [15,16,50,51]. Of note, approximately 82% of CVC-recovered yeast isolates were *C. albicans* (9/ 11) (Table 1), which also shows that other *Candida* species have the potential to cause CVC-related invasive yeast infections. Unlike endogenously acquired *Candida* species, such as *C. glabrata*, that require previous exposure to antifungals drugs to become drug-resistant, drug-resistant *C. auris* and *C. parapsilosis* can persist on the hospital environments, devices, and hands of healthcare workers and subsequently cause drug-resistant candidiasis and/or candidemia among antifungal-naïve patients [15,16,17,50,51,52,53]. It is also noteworthy that some studies have found an association between antibiotic use and the emergence of candidemia due to *Candida* species exhibiting a high minimum inhibitory concentration (MIC) and/or intrinsic resistance to fluconazole [54,55]. Furthermore, the development of invasive candidiasis is often preceded by the *Candida* colonization of the skin and mucous membrane. *Candida* colonization at multiple body sites is a strong predictor of invasive candidiasis [56]. Along the same line, the *Candida* colonization of the airway has been observed in 20% of patients after 48 h of being on mechanical ventilation, and the longer the duration of ventilation, the higher the colonization rate [3]. Up to 94% of hospitalized patients with COVID-19 receive antimicrobial agents [57,58,59], and this might further heighten the *Candida* colonization rate. Patients with sepsis or septic shock, commonly observed in severe COVID-19 patients in the ICU, may develop a leaky gut that facilitates *Candida* translocation from the GI tract into systemic circulation [39,60,61,62].

The second group of risk factors are more specifically associated with COVID-19. First, patients with severe respiratory failure associated with COVID-19 might require extracorporeal membrane oxygenation (ECMO) [63]. ECMO involves a higher number of vascular catheters (pulmonary and peripheral arterial catheters and ECMO cannula in addition to central venous catheters). ECMO is also associated with a clotting tendency that facilitates microbial pathogen (bacteria and fungus) adhesion to the catheters, as well as leukopenia that results from the sequestration of leukocytes in the lung capillaries and peripheral tissues, and adhesion to and lysis of leukocytes by ECMO circuit. ECMO cannula are often colonized by skin commensals such as *Candida* and coagulase-negative *Staphylococcus*, a condition that predisposes one to bloodstream infection. Altogether, these risk factors predispose one to systemic infection. Second, corticosteroids have been increasingly used among hospitalized patients with COVID-19 [19]. Corticosteroids have immunosuppressive effects on neutrophils, monocytesm and macrophages and predispose patients to invasive candidiasis. Lastly, whether the severe lung epithelium damage exerted by SARS CoV-2 facilitates *Candida* adherence to basement membrane causing subsequent invasive pulmonary candidiasis is not known. To date, primary *Candida* pneumonitis is considered to be rare.

## 5. Diagnosis

The diagnosis of candidemia and other forms of invasive candidiasis remains challenging, which is mostly due to the low number of yeast cells in circulation or infected tissue [64], a requirement of an invasive procedure for diagnosing deep-seated candidiasis, and the use of non-fungal-specific media to culture clinical samples [65]. While culture remains the gold standard, approximately 50% of the invasive candidiasis are not identified by blood culture, and the application of non-culture diagnostics—i.e., β-D-Glucan (BDG) and mannan antigen testing, and molecular platforms such as PCR and T2 Candida panel—are recommended to improve the diagnosis [64]. BDG (Associates of Cape Cod Diagnostics; MA, USA) is a panfungal marker and therefore a positive result is not specific for invasive candidiasis. The sensitivity and specificity for diagnosing invasive candidiasis are around 80% [66,67], and can further be increased when combined with procalcitonin, which may help to differentiate fungal from bacterial infections [68], but false positive results have been described, in particular in conditions associated with fungal translocation in the gut, such as sepsis or advanced liver cirrhosis [61,69]. BDG results should therefore be carefully evaluated and always interpreted with other clinical data. Importantly, serum BDG has been shown to be a reliable tool for antifungal stewardship, and has a high negative predictive value for invasive *Candida* infections, allowing for the early discontinuation of empirical antifungal therapy if tested from samples drawn before treatment initiation [70,71]. Enzyme-linked immunosorbent assay (ELISA) kits for the detection of *Candida* mannan antigen are commercially available to detect *Candida* in serum samples for the diagnosis of invasive candidiasis (Platelia™ Candida Ag, Bio-Rad), and are associated with a relatively high specificity and sensitivity [72]. In a recent meta-analysis, blood PCR was associated with a pooled sensitivity and specificity for proven or probable invasive candidiasis vs. at-risk controls of 95% and 92%, respectively [73]. The recently developed T2Candida Panel (T2Biosystems) combines ITS2 region amplification and T2 magnetic resonance, and can directly detect *Candida* spp. in EDTA blood samples within 5 h and has proved efficient for the diagnosis of candidemia and intra-abdominal candidiasis, although the technical demands can be a drawback [74,75,76].

Combining multiple techniques is recommended in order to improve the sensitivity of the techniques [64,77,78]. However, while serum BDG testing and screening has been used successfully in COVID-19 patients for the detection of COVID associated aspergillosis [19], the utility of other techniques remains to be determined in the context of COVID-19 patients with ARDS.

## 6. Treatment and Future Directions

Since invasive yeast infections are associated with a higher mortality in COVID-19 cases not receiving antifungal treatment compared to those receiving it [23], prompt diagnosis and treatment is of paramount importance to achieve clinical success. The management of invasive candidiasis in patients with COVID-19 is similar to that of non-COVID-19 patients. Echinocandins are the treatment of choice for invasive *Candida* infections, with fluconazole, liposomal amphotericin B, voriconazole, posaconazolem and isavuconazole being the second line alternatives [79,80,81]. Source control, including, if feasible, the removal of central venous catheters in candidemic patients, is a major determinant factor of the outcome. Echinocandins are usually well tolerated and have a favorable pharmacokinetic (PK) profile, with very few drug–drug interactions [82]. A major drawback of echinocandins is their intravenous formulation. While not impacting most hospitalized and ICU patients, it is a factor for step-down therapy or prophylaxis. The triterpenoid ibrexafungerp is a new class of structurally distinct glucan synthase inhibitors, which is currently being evaluated in various phase III trials, showing an excellent bioavailability after oral intake [83]. Moreover, the penetration of currently available echinocandins into the abdominal infection site might not be optimal, and the emergence of echinocandin-resistant *Candida* isolates during treatment, especially *C. glabrata,* is problematic [44,84]. The newer generation of echinocandins, such as the long PK and the once-weekly drug rezafungin, have shown a favorable penetration in models of IAC when compared to other echinocandins [84]. Another novel antifungal in the pipeline that will likely advance the management of invasive candida infections in the near future is fosmanogepix. It has a novel mechanism of action that inhibits the highly conserved fungal enzyme Gwt1, which is essential for the biosynthesis of glycosylphosphatidylinositol anchors.

Among patients with septic shock attributed to invasive candidiasis, the timely administration of antifungal therapy is paramount for a favorable outcome. Consistent with the data described in this overview, we need to increase our efforts to understand the full extent of this invasive fungal complication in COVID-19, and to design the best diagnosis and therapy. What should be done in the future? Since blood culture has a poor sensitivity and delayed turnaround time, the development of predictive scores or diagnostic tests that yield high positive and/ or negative predictive values is sorely needed. Diagnostics directly from blood may offer the fastest laboratory results for high-risk patients. Among COVID-19 patients, the incidence of super-infections due to *Candida* is currently not known. It is also unknown whether *Candida* super-infection leads to excess mortality or if it is merely a marker of the severity of COVID-19 infection. Well-designed and careful epidemiologic studies are needed to define the true burden of invasive candidiasis among patients with COVID-19. Prospective studies that include systematic blood and other biological sample collection might enhance future research in invasive *Candida* infections.

## Figures and Tables

**Table 1:** Clinical and microbiological features of COVID-19-associated invasive yeast infections in studies with detailed clinical and microbiological data.

Country (Case Number; %) [ref.]	Age/Sex	Underlying Conditions	Risk Factors	Hospitalization Duration	Days to Camdidemia Positivity	Species (Resistance Pattern)	Treatment	Outcome
**India (15/596; 2.5%)** [27]	25/F	CLD with grade II encephalopathy, AKD	Antibiotic use, CVC, and UC	35	14	*C. auris* (MAR), blood culture	AMB	Survived
	52/M	HT, DM	Antibiotic use, steroid therapy, CVC, and UC	20	14 and 17	*C. auris* (FLCR), blood culture	MFG and AMB	Died
	82/F	HT, DM, hypothoidism, on dialysis for CKD stage 5	Antibiotic use, steroid therapy, CVC, and UC	60	42 and 47	*C. auris* (FLCR), blood culture	MFG	Died
	86/F	CLD, IHD, DM	Antibiotic use, steroid therapy, CVC, and UC	21	10	*C. auris* (FLCR), blood culture	MFG	Died
	66/M	HT, DM, asthma	Antibiotic use, CVC, and UC	20	11 and 15	*C. auris* (FLCR+AMBR), blood culture	MFG and AMB	Survived
	71/M	Hypothoidism, on dialysis for CKD stage 5	Antibiotic use, steroid therapy, CVC, and UC	32	12 and 17	*C. auris* (FLCR), blood culture	MFG	Died
	67/M	HT, DM, COPD	Antibiotic use, steroid therapy, CVC, and UC	21	11	*C. auris* (FLCR+AMBR), blood culture	AMB and MFG	Survived
	72/M	HT, CLD	Antibiotic use, steroid therapy, CVC, and UC	27	16 and 19	*C. auris* (MAR+AMBR), blood culture	MFG	Died
	81/M	DM, HT, IHD	Antibiotic use, steroid therapy, CVC, and UC	20	15	*C. auris* (MAR), blood culture	MFG	Died
	69/M	HT, asthma	Antibiotic use, steroid therapy, CVC, and UC	21	14	*C. auris* (FLCR+AMBR), blood culture	MFG	Survived
	56/M	HT, COPD	Antibiotic use, CVC, and UC	18	7	*C. tropicalis* (S), blood culture	MFG	Survived
	69/M	HT, DM, obesity, IHD	Antibiotic use, CVC, and UC	27	8	*C. albicans* (S), blood culture	MFG	Survived
	43/F	HT	Antibiotic use, steroid therapy, CVC, and UC	24	12	*C. albicans* (S), blood culture	MFG	Survived
	47/M	Asthma, DM	Antibiotic use, CVC, and UC	18	5	*C. albicans* (S), blood culture	AMB and MFG	Died
	66/M	HT	Antibiotic use, steroid therapy, CVC, and UC	28	7	*C. krusei* (IFR), blood culture	AMB	Died
**Oman (5/ ND) ^A^** [25]	76/M	None	Antibiotic use, CVC	ND	ND	*C. albicans* (S), CVC culture	No	Died
	68/M	HT	Antibiotic use, CVC	ND	ND	*C. albicans* (S), blood culture	CAS + CVC removal	Died
	38/M	HT, dyslipidemia, old stroke	Antibiotic use, CVC	ND	ND	*C. glabrata* (S), blood culture	MFG→CAS→AMB	Died
	64/M	HT	Antibiotic use, CVC	ND	ND	*C. albicans* + *C. tropicalis* (S), blood culture	CAS+VRC	Survived
	49/M	None	Antibiotic use, CVC	ND	ND	*C. albicans* (S), blood culture	CAS	Survived
**UK (17/135; 12.6%)** [23]	ND	HT, obesity	CVC	ND	ND	No ID, CVC	FLC	Died
	ND	HT		ND	ND	*Rhodotorula* spp., blood culture	CAS, LAMB	Died
	ND	Oseophagectomy, cancer	Hydrocortisone	ND	ND	No ID, from chest drain	FLC	Died
	ND	Ulcerative colitis	CVC	ND	ND	*C. albicans*, CVC	None	Survived
	ND	DM, HT, obesity, asthma	CVC	ND	ND	*C. albicans*, CVC	FLC	Survived
	ND	HT, asthma		ND	ND	*C. albicans*, blood culture, BDG = 156, 95, 86, *Candida* PCR = Positive	CAS	Survived
	ND	Haem, cardiac		ND	ND	*C. albicans*, blood culture	None	Died
	ND	None	CVC	ND	ND	*C. albicans*, CVC	FLC	Survived
	ND	Cardiac, CKD, cancer	CVC	ND	ND	*C. albicans*, CVC	CAS	Died
	ND	Asthma, inflammatory, irritable bowel syndrome	CVC	ND	ND	*C. albicans*, CVC	VRC	Died
	ND	None	CVC	ND	ND	*C. parapsilosis*, CVC	CAS	Survived
	ND	None	CVC	ND	ND	*C. albicans*, CVC and blood culture	FLC	Died
	ND	None		ND	ND	*C. albicans*, blood culture, BDG > 500, *Candida* PCR = Positive	FLC, CAS	Survived
	ND	DM, HT, Obesity	CVC	ND	ND	*C. albicans*, CVC	FLC	Survived
	ND	Hepatitis, intravenous drug user, neutropenia, cellulitis		ND	ND	*C. albicans* + *C. parapsilosis*, blood culture, BDG = 386	FLC, LAMB	Survived
	ND	DM, inflammatory, alcoholic	Steroid therapy (ND)	ND	ND	*C. albicans*, ascites culture	CAS, VRC	Survived
	ND	DM, HT		ND	ND	*C. albicans*, CVC, BDG > 500	FLC, VRC	Died
**Italy (3/43; 8%)** [26]	67/M	Cerebral ischemia	Parentral nutrition, antibiotic use, CVC, and Tocilizumab (8 mg/kg)	ND	13	*C. albicans*, blood culture	CAS+FLC	Survived
	58/M	HT	Parenteral nutrition and Tocilizumab (8 mg/kg)	ND	17	*C. tropicalis*, blood culture	CAS	Survived
	78/M	DM and obesity	Parenteral nutrition, antibiotic use, steroid therapy, CVC, and Tocilizumab (8 mg/kg)	ND	13	*C. parapsilosis*, blood culture	CAS+FLC	Survived
**Italy (1/ND)** [24]	79/M	DM, IHD, stadium IV peripheral artery disease	Antibiotic use and surgery	35	53	*C. glabrata* (PER), blood culture	CAS	Died
**Greece (2/ND)** [21]	76/M	HT	Antibiotic use, Ultra-Levure (250–500 mg/day)	80	35 (4 days after Ultra-Levure)	*S. cerevisiae* (S), blood culture	AND→FLC	Survived
	73/M	HT and DM	Antibiotic use, Ultra-Levure (250–500 mg/day)	Transferred to a regional hospital	15 (6 days after Ultra-Levure)	*S. cerevisiae* (S), blood culture	AND→FLC	Survived

A. The total number of severely ill patients was not determined for studies from Italy [24], Greece [21], and Oman [25]. AKD: Acute kidney disease; CKD: Chronic kidney disease; CLD: Chronic liver disease; COPD: Chronic obstructive pulmonary disease; DM: Diabetes mellitus; HT: Hypertension; IHD: Ischemic heart diseases; CVC: Central venous catheter; UC: Urinary catheter; AMB: Amphotericin B; LAMB: Liposomal AMB; MFG: Micafungin; CAS: Caspofungin; VRC: Voriconazole; FLC: Fluconazole; S: Susceptible; FLCR: Fluconazole-resistant; AMBR: Amphotericin B-resistant; IFR: Intrinsically fluconazole-resistant MAR: Multiazole-resistant; MDR: Multidrug-resistant; PER: Pan-echinocandin-resistant; ND: Not determined; PCR: Polymerase chain reaction; BDG: Beta-d-glucan.
